# CXCL12/CXCR4 axis supports mitochondrial trafficking in tumor myeloma microenvironment

**DOI:** 10.1038/s41389-022-00380-z

**Published:** 2022-01-21

**Authors:** Cesarina Giallongo, Ilaria Dulcamare, Daniele Tibullo, Vittorio Del Fabro, Nunzio Vicario, Nunziatina Parrinello, Alessandra Romano, Grazia Scandura, Giacomo Lazzarino, Concetta Conticello, Giovanni Li Volti, Angela Maria Amorini, Giuseppe Musumeci, Michelino Di Rosa, Francesca Polito, Rosaria Oteri, M’hammed Aguennouz, Rosalba Parenti, Francesco Di Raimondo, Giuseppe A. Palumbo

**Affiliations:** 1 Department of Medical, Surgical Sciences and Advanced Technologies G.F. Ingrassia, University of Catania, 95123 Catania, Italy; 2 Division of Hematology, AOU Policlinico, 95123 Catania, Italy; 3 Department of Biomedical and Biotechnological Sciences, Section of Biochemistry, University of Catania, 95123 Catania, Italy; 4 Department of Biomedical and Biotechnological Sciences, Section of Physiology, University of Catania, 95123 Catania, Italy; 5 Department of General Surgery and Medical-Surgical Specialties, University of Catania, 95123 Catania, Italy; 6 UniCamillus - Saint Camillus International University of Health Sciences, 00131 Rome, Italy; 7 Department of Biomedical and Biotechnological Sciences, Human, Histology and Movement Science Section, University of Catania, 95123 Catania, Italy; 8 Department of Clinical and Experimental Medicine, University of Messina, 98122 Messina, Italy

**Keywords:** Cancer microenvironment, Cancer metabolism

## Abstract

Mesenchymal stromal cells (MSCs) within the protective microenvironment of multiple myeloma (MM) promote tumor growth, confer chemoresistance and support metabolic needs of plasma cells (PCs) even transferring mitochondria. In this scenario, heterocellular communication and dysregulation of critical signaling axes are among the major contributors to progression and treatment failure. Here, we report that myeloma MSCs have decreased reliance on mitochondrial metabolism as compared to healthy MSCs and increased tendency to deliver mitochondria to MM cells, suggesting that this intercellular exchange between PCs and stromal cells can be consider part of MSC pro-tumorigenic phenotype. Interestingly, we also showed that PCs promoted expression of connexin 43 (CX43) in MSCs leading to CXCL12 activation and stimulation of its receptor CXCR4 on MM cells favoring protumor mitochondrial transfer. Consistently, we observed that selective inhibition of CXCR4 by plerixafor resulted in a significant reduction of mitochondria trafficking. Moreover, intracellular expression of CXCR4 in myeloma PCs from BM biopsy specimens demonstrated higher CXCR4 colocalization with CD138+ cells of non-responder patients to bortezomib compared with responder patients, suggesting that CXCR4 mediated chemoresistance in MM. Taken together, our data demonstrated that CXCL12/CXCR4 axis mediates intercellular coupling thus suggesting that the myeloma niche may be exploited as a target to improve and develop therapeutic approaches.

## Introduction

Multiple myeloma (MM) is a malignancy of plasma cells (PCs) that accumulate within the bone marrow (BM) and is associated with monoclonal protein in serum and/or urine. Myeloma tumor cells can also be detected in extramedullary sites and/or peripheral blood during progression of disease [1]. Previous reports showed that BM microenvironment plays a crucial role in sustaining tumor cell growth and disease progression, underling the complex interaction between myeloma PCs and their milieu [2, 3]. Mesenchymal stromal cells (MSCs) constitute the main compartment of BM stroma and have shown to support, independently from intrinsic PC factors, the progression from the premalignant monoclonal gammopathy of undetermined significance (MGUS) and/ or smoldering multiple myeloma (SMM) to malignant MM [4]. We and others have previously described a significant different phenotype of MM-MSCs compared to MSC from healthy donors [5–8], according to their remodeling in a pathological niche. To this regard, it has been demonstrated that interferon-responsive effector T cell and CD8+ stem cell memory T cell populations release proinflammatory cytokines activating myeloma-specific inflammatory MSCs [9]. These inflammatory stromal cells colocalize with tumor PCs and their gene signature promotes myeloma survival and impact on the immune system functioning. Consistently, Schinke and colleagues showed that gene expression patterns of MM-MSCs have an independent prognostic impact on survival and is predictive of myeloma patient outcomes [4]. Finally, chronic inflammation within the tumor niche leads MM-MSCs to activate TLR4 signaling, which polarizes them toward a pro-tumorigenic phenotype, favoring immunosuppression and tumor growth [7].

Metabolic rewiring is also involved in the regulation of MSC phenotype and, after stimulation by pro-inflammatory mediators, the immunosuppressive ability of MSCs is sustained by a glycolytic metabolism which is required to support secretion of immunosuppressive factors [10]. To this regard, the consequent reduction of mitochondria metabolism may have an impact on intercellular communication. Indeed, MSCs have been shown to donate their mitochondria within the BM niche and such a trafficking has a role in adapting metabolic processes in both non-malignant and malignant cells [11–14]. In this context, several processes have been described including the formation of tunneling nanotubes (TNT), the release of extracellular vesicles (EVs), and gap junction formation [12, 14, 15]. Interestingly, transferred mitochondria from MSCs to tumor cells are functionally active and lead to protection against tissue injury or resistance to therapeutic agents [14, 16]. Consistently, acute myeloid leukemia (AML) blasts use mitochondria uptake to acquire a metabolic advantage [11] and thus being less sensitive to mitochondrial depolarization after chemotherapy [13]. Furthermore, the transfer of mitochondria from MSCs to myeloma PCs mediates tumor proliferation by promoting mitochondrial-based oxidative phosphorylation by a CD38-dependent mechanism that supports the formation of TNT [12]. Cell to cell contact is also involved for both mitochondrial transfer and increased ATP levels. To this regard, recent studies described the involvement of the mitochondrial ρ-GTPase Miro1 [17] or the gap junction-forming protein connexin 43 (CX43) [18]. Interestingly, the expression of CX43 is increased in MM-MSCs and enhances secretion of the chemokine CXCL12 (also known as stromal-derived factor 1; SDF-1) [19]. CXCL12 is the ligand of CXCR4 and mediates the homing mechanisms of both healthy and myeloma PCs into the BM. Moreover, it plays an important role in the retention of malignant PCs in the BM niche, increasing their adhesion to MSCs and promoting PC survival and chemoresistance [19].

The aim of the present study was to determine if the MM-MSC phenotype is associated to a higher mitochondrial trafficking and assess whether mitochondria uptake is regulated by CXCL12/CXCR4 axis in order to provide new potential treatment strategy to overcome chemoresistance and improve clinical outcome and quality of life of MM patients.

## Material and methods

### Cell cultures and treatments

Human MM cell lines (HMCLs) were cultured in RPMI-1640 with 10% (MM1S, H929, OPM2) or 20% (U266) FBS and 1% penicillin-streptomycin. Commercially available stromal cell lines HS-5 were grown in DMEM supplemented with 10% FBS. After written informed consent (Azienda ospedaliero Universitaria Policlinico-Vittorio Emanuele, #34/2013/VE), MSCs were isolated from BM samples collected from MM patients at diagnosis and age-matched healthy controls. BM mononuclear cells were obtained after density gradient centrifugation on Ficoll and cultured in DMEM supplemented with 10% FBS. After 3 days culture, non-adherent cells were removed, whereas MSCs were selected by their adherence to the plastic plates. Cultures were maintained at 37 °C and 5% CO_2_ and expanded until the third or fourth passage.

Ioxynil octanoate (IO) was purchased from Riedel-de Haen (Switzerland). Plerixafor, bortezomib, carfilzomib, and verapamil were obtained from Sigma-Aldrich.

### Primary CD138+ cells isolation

After centrifugation of BM samples on ficoll, cells were purified using CD138 microbeads according to the manufacturer’s instructions (Miltenyi Biotech, Bologna, Italy), tested for purity using flow cytometry, and then used for in vitro experiments.

### HPLC

Metabolic analysis was driven after deproteinization of cell samples according to a protocol suitable to obtain protein-free extracts for further HPLC analysis of acid labile and easily oxidizable compounds. After washing with PBS at pH 7.4, cell pellets were deproteinized adding 1 mL of ice-cold, nitrogen-saturated, CH3CN + 10 mM KH2PO4, pH 7.4 (3:1, v-v). After vigorous mixing for 60 s, samples were centrifuged at 20,690 × *g* for 10 min at 4 °C. The organic solvent was removed from the deproteinizing mixture using two washings with 5 mL of chloroform. The upper aqueous phase obtained by centrifugation at the same conditions was then used for the HPLC analysis of low molecular weight metabolites.

Simultaneous separation of low molecular weight metabolites related to energy metabolism, including high energy phosphates (ATP, ADP, AMP), oxidized and reduced nicotinic coenzymes (NAD+, NADH), was carried out using a Hypersil C-18, 250 × 4.6 mm, 5 µm particle size column, provided with its own guard column (Thermo Fisher Scientific, Rodano, Milan, Italy), following previously established ion pairing HPLC methods [20]. The HPLC apparatus was based on a SpectraSYSTEM P4000 pump (Thermo Fisher Scientific) interfaced to a highly sensitive UV6000LP diode array detector (Thermo Fisher Scientific), equipped with a 5 cm light path flow cell and set up between 200 and 300 nm wavelength. Assignment and calculations of the aforementioned compounds in cell extracts, were performed by comparing retention times, absorption spectra, and area of the peaks (calculated at 260 nm wavelength for all compounds) of chromatographic runs of mixtures containing known concentrations of ultrapure standards. Energy charge potential (ECP) was calculated as follows: ATP+1/2 ADP/ATP+ADP+AMP.

### Determination of intracellular lactate levels

After deproteinization, samples were added to a mixture containing 100 mM Tris-HCl, 1.5mM N-ethyl-N-2-hydroxy-3-sulfopropyl-3-methylalanine, 1.7 mM 4-aminoantipyrine, and 5 IU horseradish peroxidase [21]. The reaction was started with the addition of 5 IU of lactate oxidase. Using an Agilent 89090A spectrophotometer (Agilent Technologies, Santa Clara Ca, USA), the absorbance at 545 nm wavelength of each sample was interpolated with a calibration curve obtained by plotting absorbance measured in standard lactate solutions with increasing known concentrations. Intracellular lactate levels were normalized with respect to the number of cells and expressed as nmol/10^6^ of cells.

### Measurement of extracellular acidification (ECAR)

The level of ECAR was determined by the Seahorse Extracellular Flux Analyzer XF24 (Seahorse Bioscience/ Agilent Milan Italy) in HC-MSCs (*n* = 3) and MM-MSCs (*n* = 3). 3 × 10^4^ cells were cultured in the XF24-well plate overnight. Then, cells were sequentially cultured with glucose, oligomycin (oxidative phosphorylation inhibitor), and 2-deoxy-D-glucose (2-DG) glycolytic inhibitor, according to the manufacturer’s instructions and protocols. Seahorse XF-24 Wave software was used to analyze the data and ECAR detection was represented as mpH/min.

### Analysis of mtDNA

Total genomic DNA was isolated using the PureGene kit (Qiagen, Hilden, Germany) according to the manufacturer’s instructions. The expression of mtDNA tRNALeu (UUR) (forward: 5′-CACCCAAGAACAGGGTTTGT-3′ and reverse: 5′-TGGCCATGGGTATGTTGTTA-3′) was determined by qPCR using Brilliant III Ultra-Fast SYBR Green QPCR Master Mix. β2-microglobulin (forward: 5′-TGCTGTCTCCATGTTTGATGTATCT-3′ and reverse: 5′-TCTCTGCTCCCCACCTCTAAGT-3′) was used to determine nuclear DNA (nDNA) as an internal control [22]. qPCR was performed in triplicate for each DNA sample. mtDNA/nDNA ratio was calculated and graphed [23].

### MitoTracker-based mitochondrial transfer assay by flow cytometry

Human primary MSCs or HS-5 cells were stained with 200 nM MitoTracker Red CMXRos probe (Thermo Fisher Scientific, Milan, Italy) for 30 min at 37 °C, according to the manufacturer’s instructions. In the set of experiments with drugs added to co-cultures, HMCLs were also stained with 200 nM MitoTracker Red CMXRos probe. Then, cells were washed 3 times in phosphate buffered saline (PBS) to remove the unbound probe. HMCLs or primary PCs were co-cultured with MSCs at a 5:1 ratio. PCs was also grown in monoculture as control. After 24 h MM cells were removed from coculture and MitoTracker fluorescence was analyzed by flow cytometer MACSQuant Analyzer 10 (Miltenyi Biotec, Bologna, Italy) or OPERETTA platform (PerkinElmer, Monza, Italy). Flow cytometric analysis was performed gating on CD45^+^ cells to exclude contaminating MSCs (Supplementary Fig. 1) and doublets were excluded using FSH-A and FSH-H. This assay was used to quantify mitochondrial transfer calculating the difference in MitoTracker fluorescence between MM cells grown with and without MSCs.

Operetta platform was used to quantify mitochondrial transfer in time lapse. In this case, HMCLs were labeled with 1 µM Calcein AM (Thermo Fisher Scientific, Monza, Italy) for 30 min at 37 °C. After 3 washes, PCs were added to wells with MitoTracker red-stained HS-5 cells. HMCLs were also grown in monoculture for 24 h as a control. Then, the plate was incubated into Operetta and maintained at 37 °C. Images were captured at intervals (every 3 h). The data were analyzed by Harmony software (Perkinelmer).

### Immunofluorescence

After co-culture, cells were fixed using 4% paraformaldehyde, permeabilized using 0.1% Triton X, and blocked to prevent nonspecific antibody binding using 2% bovine serum albumin [24]. The slides were then incubated overnight at 4 °C with the primary antibodies mouse anti-CXCL12 (MA5-23759, Thermo Fisher Scientific) and rabbit anti-CX43 (#3512, Cell Signaling Technology, Danvers, MA, USA) at 1:100 dilution. Subsequently, cells were washed three times in PBS for 5 min and then incubated for 1 h at room temperature with the appropriate combination of fluorescence conjugated secondary antibodies goat polyclonal anti-mouse Alexa Fluor 546 (A21045, Thermo Fisher Scientific) and goat polyclonal anti-rat Alexa Fluor 647 (A21247, Thermo Fisher Scientific) at 1:1000 dilution. Phalloidin staining was performed using Phalloidin-iFluor 488 (ab176753, Abcam, Milan, Italy) at dilution 1:500 and incubated for 1 h at room temperature. Samples were then washed in 0.1% Triton X-100 in PBS and nuclei were counterstained with DAPI (1:1000) for 5 min, at room temperature. Slices were mounted with fluorescent mounting medium Permafluor (Thermo Fisher Scientific) and digital images were acquired using a Leica DM IRB (Leica Microsystem, Buccinasco, Milan, Italy) fluorescence microscope or a Leica TCS SP8 confocal microscope.

To evaluate mitochondrial transfer by immunofluorescence, we established GFP-expressing U266 cell lines by transfecting GFP expression plasmids to distinguish MM cells from MitoTracker stained MM-MSCs. For primary CD138+ cells Calcein AM staining was used.

Paraffin sections from MM patients BM were deparaffinized and rehydrated as previously described [25]. After permeabilization and blocking as described above, sections were stained overnight at 4 °C with the following primary antibodies: rabbit anti-CD138 (AB128936, ABCAM) and goat anti-CXCR4 (GTX21670, Genetex, Irvine, CA, US). After washing, secondary staining was performed for 1 h at room temperature using donkey polyclonal anti-goat Alexa Fluor 647 (A32849, Thermo Fisher Scientific; 1:200) and subsequently with goat polyclonal anti-rat Alexa Fluor 647 (A21247, Thermo Fisher Scientific). DAPI was used as nuclear stain. The fluorescent images were obtained using a Zeiss Axio Imager Z1 Microscope with Apotome 2 system (Zeiss, Milan, Italy).

### qPCR

After RNA extraction, reverse transcription was performed by using the High Capacity cDNA Reverse Transcription Kit (Thermo Fisher Scientific). Then the relative transcription of human gene CXCL12 (Fw: TCAGCCTGAGCTACAGATGC; Rw: CTTTAGCTTCGGGTCAATGC) was determined by RTqPCR using Brilliant III Ultra-Fast SYBR Green QPCR Master Mix (Agilent Technologies, Milan, Italy) and 7900HT Fast Real-Time PCR System (Thermo Fisher Scientific). For each sample, the relative expression level of the mRNA of interest was determined by comparison with the control housekeeping genes B2M (Fw: AGCAGCATCATGGAGGTTTG; Rw: AGCCCTCCTAGAGCTACCTG) and GAPDH (Fw: AATGGGCAGCCGTTAGGAAA; Rw: GCCCAATACGACCAAATCAGAG) using the 2^^−ΔΔCt^ method.

### Western blot

For Western blot analysis 30 μg of protein was loaded onto a 12% polyacrylamide gel Mini- PROTEAN TGXTM (BIO-RAD, Milan, Italy) followed by electrotransfer to nitrocellulose membrane Trans-Blot TurboTM (BIO-RAD, Mylan, Italy) using Trans-Blot SE Semi-Dry Transfer Cell (BIO-RAD). Subsequently, membrane was blocked in chemiluminescent blocker (Millipore, Darmstadt, Germany) for 1 h at room temperature. After blocking, the membrane was three times washed in PBS for 5 min and incubated with primary antibodies against human CXCL12 (MA5-23759, Thermo Fisher Scientific), CX43 (#3512, Cell Signaling Technology, Danvers, MA, USA), and β-actin (ab181602, Abcam). Next day, after three washes in TBST, the membranes were incubated with antimouse (1:3000, Jackson, WestGrove, PA, USA) and anti-rabbit HRP-conjugated (1:3000, Jackson, WestGrove, PA, USA) secondary antibodies for 1 h at RT. Proteins bands were visualized with premixed ready-to-use chemiluminescent HRP detection reagent (Millipore) according to the manufacturer’s instructions and captured using the *C*-*DiGit Blot Scanner(LI*-*COR Biosciences*, Nebraska USA). The density of each band was quantified using ImageJ analysis software and normalized β-actin levels measured in the same membranes.

### Flow cytometry analyses of CXCR4 expression

CXCR4 expression was evaluated by flow cytometry using anti-human CXCR4-PE antibody (Clone # 12G5, BECKMAN COULTER, Mylan, Italy) on a Beckman Coulter FC-500 flow cytometer. Clinical data of patients included in this study are provided in Table 1.

**Table 1:** Baseline clinical characteristics of MM patients included in the study.

	MM patients (*n* = 48)
Median age (range)	72 (39–87)
Males	54.2 (26)
Females	45.8% (22)
Stage ISS, *n*	
I	33% (16)
II	33% (16)
III	33% (16)
Newly diagnosed pts	62,5% (30)
Resistant/refractory pts	37,5% (18)
Resistant/refractory patients	
IP-resistant/refractory	100% (18)
Double-resistant/refractory (PIs and IMiDs)	44% (8)

IMiDs: immunomodulatory drugs (Lenalidomide and Pomalidomide).

### Statistical analysis

All statistics were performed using GraphPad Prism (version 5.00 for Mac, GraphPad Software, San Diego, CA, USA). All data were tested for normality using Shapiro–Wilk test. Data that passed normality test were statistical analyzed using Student’s t-test or ANOVA test where appropriate. Mann-Whitney test was used to compare datasets that were not normal distributed. A *p*-value < 0.05 was considered to indicate a statistically significant difference between experimental and control groups.

## Results

### MM-MSCs exhibit reduced reliance on mitochondrial metabolism and transfer more mitochondria to myeloma PCs

**Fig. 1:**
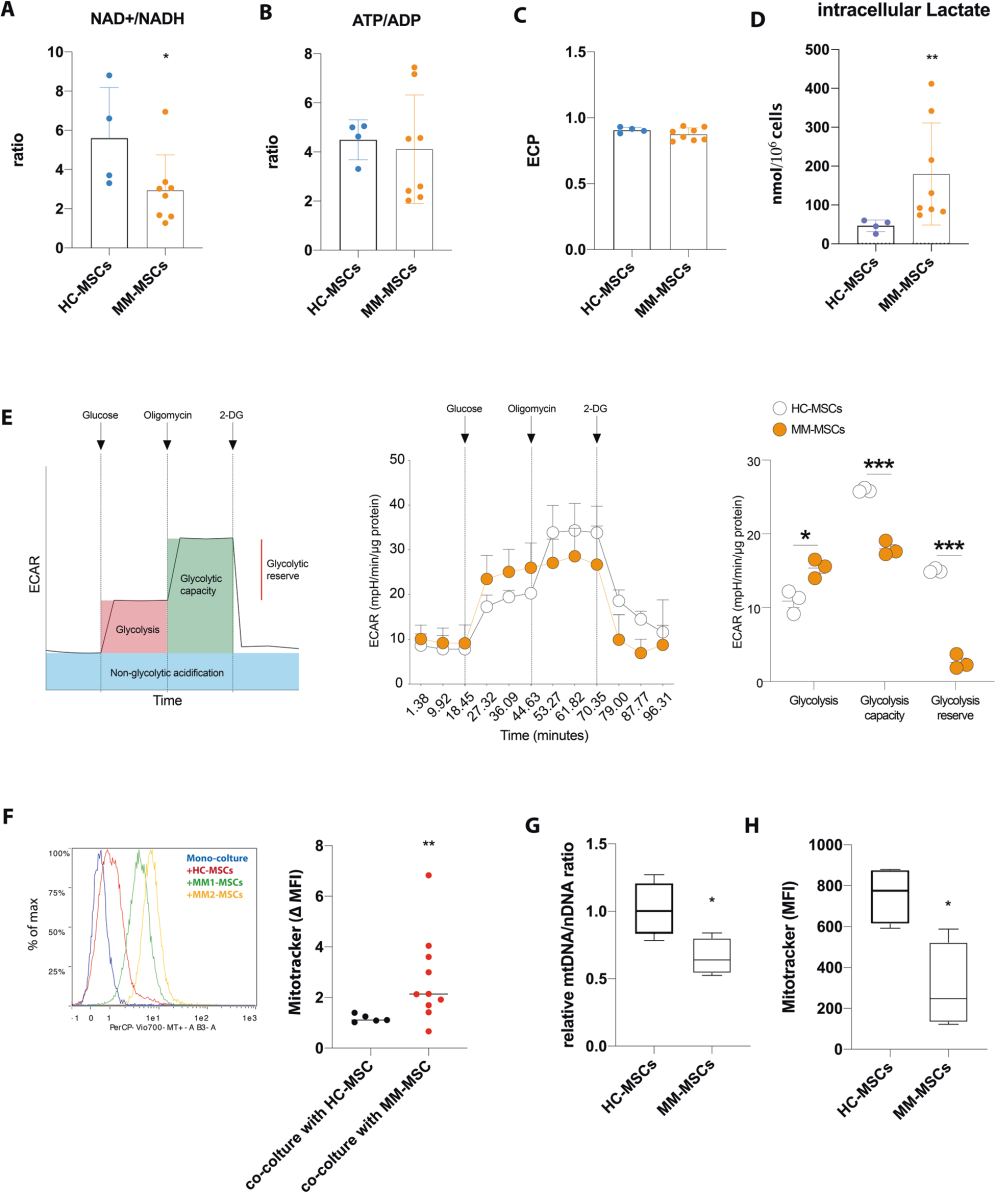
MM-MSCs are less dependent on mitochondrial metabolism and transfer more mitochondria to myeloma PCs compared to HC-MSCs. **A**–**C** NAD^+^, NADH, ATP and ADP concentrations were calculated by HPLC analysis in deproteinized HC-MSCs (*n* = 4) and MM-MSCs (*n* = 8) and ratio were graphed. ECP was calculated as follows: ATP+1/2 ADP/ATP+ADP+AMP. **D** Intracellular lactate concentration was measured by enzymatic assay **E** Representative extracellular acidification rate (ECAR) plot during glycolytic stress test showing non-glycolytic acidification, glycolysis, glycolysis capacity and glycolytic reserve and representative plots of HD-MSCs (*n* = 3) and MM-MSCs (*n* = 3) upon glucose, oligomycin, and 2-DG exposition over time. **F** MitoTracker Red CMXRos uptake by U266 cells (CD45+ gated) was measured by flow cytometry after 24 h co-culture with MitoTracker labeled HC-MSCs (*n* = 5) or MM-MSCs (*n* = 10). Δ MitoTracker-mean fluorescence intensity (calculated as the difference between red autofluorescence of U266 cells grown alone and MitoTracker fluorescence of the same cells co-cultured with MSCs) was graphed. A representative flow cytometry histogram shows the comparison of MitoTracker red fluorescence of U266 cells in monoculture vs cells in coculture with HC- or MM-MSCs. **G**, **H** Box plots showing mitochondrial content of HC-MSCs (*n* = 4) and MM-MSCs (*n* = 4). **F** Relative quantification of mtDNA levels was performed using qPCR; **G** mitochondrial mass was evaluated by flow cytometry after MitoTracker staining. Bars indicate the standard error means (*p < 0.05; **p < 0.01; ***p < 0.001).

To test our hypothesis of a metabolic rewiring of MM-MSCs, we analyzed metabolic profile of healthy control (HC-) and MM-MSCs. Figure 1A, B shows that NAD^+^/NADH ratio was decreased in myeloma MSCs (*n* = 8) as compared with HC-MSCs (*n* = 4, *p* < 0.05), meanwhile ATP/ADP ratio was not significantly different between the two groups. This different metabolic phenotype showed by MM-MSCs was not related to alterations of the energy state as suggested by similar values of ECP observed in HC- and MM-MSCs (Fig. 1C). Also, intracellular lactate concentration resulted significantly higher in MM-MSCs (*p* < 0.01; Fig. 1D), suggesting an increased glycolytic profile (Fig. 1A–D). We then moved to confirm this observation using a seahorse-assisted glycolysis assessment. Our data shows that MM-MSCs exhibited significantly increased glycolytic rate (Fig. 1E). The metabolic rewiring of MM-MSCs could be dependent on hypoxic BM microenvironment, the niche which gives rise to these cells. Indeed, HS-5 cells cultured for 24 h in hypoxic conditions showed similar metabolic features showed by MM-MSCs as suggested by lower NAD^+^/NADH ratio and higher intracellular lactate as compared to normoxic cells (Supplementary Fig. 2).

This led us to analyze whether MM-MSCs were much prone in transferring mitochondria than HC-MSCs. We first labeled HC- and MM-MSCs with Mitotracker Red CMXRos before co-culture with U266 cells. After 24 h of coculture, we quantified mitochondria transfer by flow cytometry comparing the difference between red autofluorescence of U266 cells grown alone and MitoTracker fluorescence of the same cells co-cultured with HC- or MM-MSCs (Δ MitoTracker-mean fluorescence intensity, ΔMFI). The obtained values were significantly higher in U266 co-cultured with MM-MSCs (*n* = 10) as compared to PCs co-cultured with HC-MSCs (*n* = 5) (ΔMFI values: 2.7 ± 1.7 vs 1.2 ± 0.1; *p* < 0.01; Fig. 1F). To exclude that this higher mitochondrial trafficking from MM-MSCs to PCs could be linked to a higher mitochondrial content of MM-MSCs as compared to HC-MSCs, we evaluated mtDNA copy number in these primary cells. As shown in Fig. 1G, the relative mtDNA/nDNA ratio was decreased in MM-MSCs (*p* < 0.05). Such evidence was coupled with a significant reduction of mitochondrial mass, assessed by MitoTracker staining (*p* < 0.05; Fig. 1H).

All these data demonstrate that MM-MSCs are relatively independent on mitochondria metabolism and are inclined to transfer more mitochondria to MM tumor cells.

### Myeloma PC-induced CX43 regulates CXCL12 production and mitochondrial transfer in MSCs

**Fig. 2:**
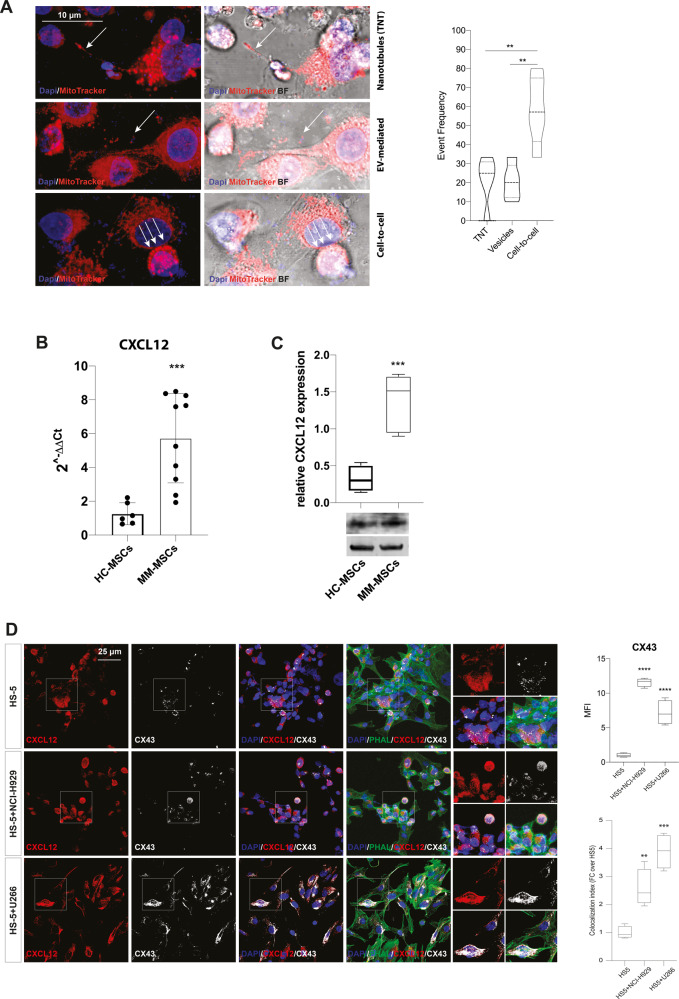
Myeloma PCs activate expression of CXCL12 and CX43 in HS-5 cell line. **A** Representative confocal microscopy images show that mitochondria were transferred to U266 cells via Tunneling Nanotubes (TNT), extracellular vesicles (EV), and cell-to-cell contact. Bar graphs show the % of events mediated by TNT, EVs, or cell-to-cell contact over the total number of transfer events observed. **B** mRNA expression of CXCL12 was evaluated in HC-MSCs (*n* = 6) and MM-MSCs (*n* = 10). **C** Western blot analysis of CXCL12 expression in MSC from HC- and MM-patients. β-actin protein was used as total protein loading reference. For analysis, the optical density of the bands was measured using Scion Image software. **D** The left panel shows representative pictures of HS-5 cells cultured alone or with NCI-H929 or U266 cell lines; cells were stained for CXCL12 (red) and CX43 (white). Quantification of CX43 MFI and of the colocalization index was reported. Bars indicate the standard error means (**p < 0.01; ***p < 0.001; ****p < 0.0001).

As shown in Fig. 2A by confocal imaging, mitochondria can be transferred from MSCs to U266 cells via TNTs, EVs, or direct cell-to-cell contact. Analyzing the frequency of events mediated by TNTs, EVs, or cell-to-cell contact over the total number of transfer events observed, cell-to-cell contact resulted the most common mechanism versus TNTs- and EVs-dependent transfer (*p* < 0.01; Fig. 2A). In accordance with these data, we found mitochondrial transfer to be significantly decreased when cell-to-cell contacts between PCs and MSCs were impeded (*p* < 0.001; Supplementary Fig. 3). As MSCs possess xenobiotic efflux pumps that may extrude MitoTracker [26], we incubated labeled HS-5 cells with verapamil (VP) and MitoTracker fluorescence was analyzed after 24 h by flow cytometry (Supplementary Fig. 4A). MitoTracker-MFI was higher in cells cultured in presence of VP. Although inhibition of efflux pumps increased the amount of MitoTracker within stromal cells, the amount of mitochondria transferred to PCs did not change even in presence of VP (Supplementary Fig. 4B), indicating that analyzed MitoTracker-MFI in MM cells is not significantly pumped out by xenobiotic efflux pumps.

**Fig. 3:**
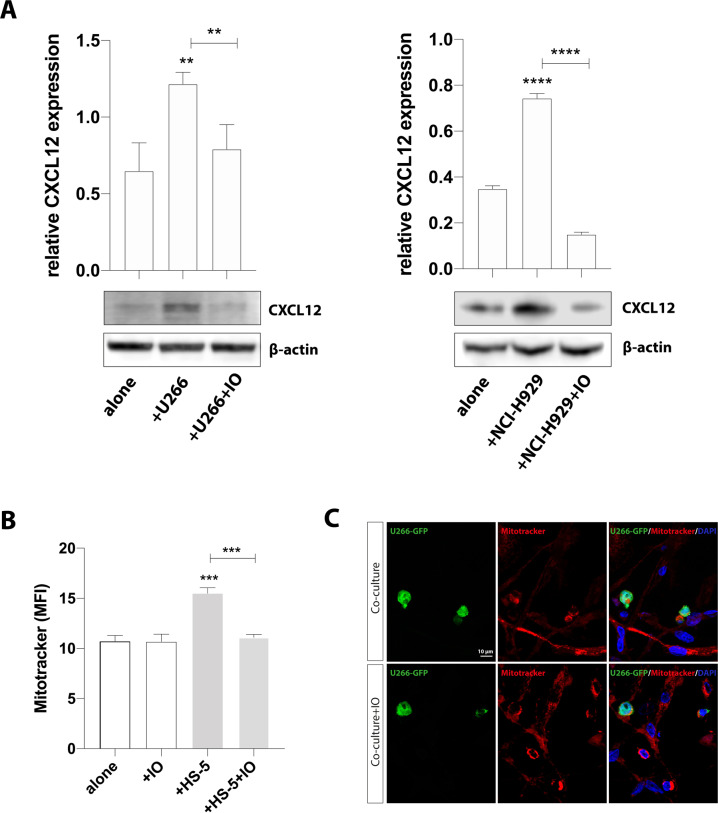
CX43 regulates PC-induced expression of CXCL12 in HS-5 cells and mediates mitochondrial transfer from MSCs to MM cells. **A** Western blot analysis of CXCL12 expression in HS-5 cells cultured alone or with MM cells in presence or not with IO. β-actin protein was used as total protein loading reference. For analysis, the optical density of the bands was measured using Scion Image software. **B** MitoTracker labeled U266 and HS-5 cells were co-cultured in presence or not of IO for 24 h. MitoTracker red fluorescence was measured in U266 cells (CD45+ gated) by flow cytometry. U266 cells cultured alone were used as control. **C** Representative images show that inhibition of CX43 decreased the uptake of mitochondria (red) from MM-MSCs by U266-GFP cells (green) after 24 h of co-culture. The data are presented as means ± SD of three independent experiments (**p < 0.01; ***p < 0.001; ****p < 0.0001).

In the cell-to-cell contact, the gap junction-forming protein CX43 has been found critical for mitochondria uptake in lung and brain injury [27, 28] and it also acts as a regulator of CXCL12 secretion by MSCs [29]. We found that MM-MSCs showed a significantly upregulated CXCL12 expression as compared to HC-MSCs (Fig. 2B, C). Therefore, we moved to co-culture HS-5 cells with U266 or NCI-H929 myeloma cell lines to evaluate whether tumor cells were able to increase the levels of both CXCL12 and CX43 in healthy MSCs. This set of experiments showed that HS-5 cells, after 24 h of coculture with either U266 or NCI-H929 cell lines, showed significantly increased CX43 expression levels (*p* < 0.0001; Fig. 2D) and CXCL12-CX43 colocalization (*p* < 0.001 and *p* < 0.01 for U266 and NCI-H929, respectively, as compared to HS-5 alone; Fig. 2D). Upregulation of CX43 after co-culture with MM cells was also confirmed by western blot (Supplementary Fig. 5). To evaluate the selective PC-induced activation of CXCL12 expression via CX43 in MSCs, we co-cultured HS-5 cells with MM cell lines and exposed cocultures to 10 µM ioxynil octanoate (IO), a selective inhibitor of CX43-based gap junctions [30]. We found that the up-regulation of CXCL12 induced by MM cells was reverted by exposition to the CX43 inhibitor (*p* < 0.01 and *p* < 0.001 for HS-5 and U266 and HS-5 and NCI-H929 cocultures treated with IO, versus control cocultures respectively; Fig. 3A), thereby indicating that CX43 activated by PCs regulates CXCL12 production in MSCs.

Given that CX43 is involved in mitochondria trafficking, we subsequently evaluated whether its inhibition decreased mitochondria uptake in tumor PCs. U266 and HS-5 cells were labeled with MitoTracker and mitochondrial transfer was evaluated after 24 h co-culture by flow cytometry. We observed that MitoTracker MFI increased in U266 cocultured with HS-5 in absence of IO (from 10.7 ± 0.5 to 15.5 ± 0.6; *p* < 0.001; Fig. 3B). The effect was abolished by CX43-base channels inhibitor IO (*p* < 0.001 versus control cocultures and *p* > 0.05 versus U266 alone, Fig. 3B).

We next confirmed these data co-culturing MitoTracker-labeled MM-MSCs with U266-GFP cell line in presence or absence of IO for 24 h and the mitochondrial transfer was detected by immunofluorescence. As shown in Fig. 3C, the incorporation of MitoTracker-labeled mitochondria in U266-GFP cells was reduced by IO treatment.

### CXCR4 expression levels correlate with mitochondrial uptake in myeloma PCs

**Fig. 4:**
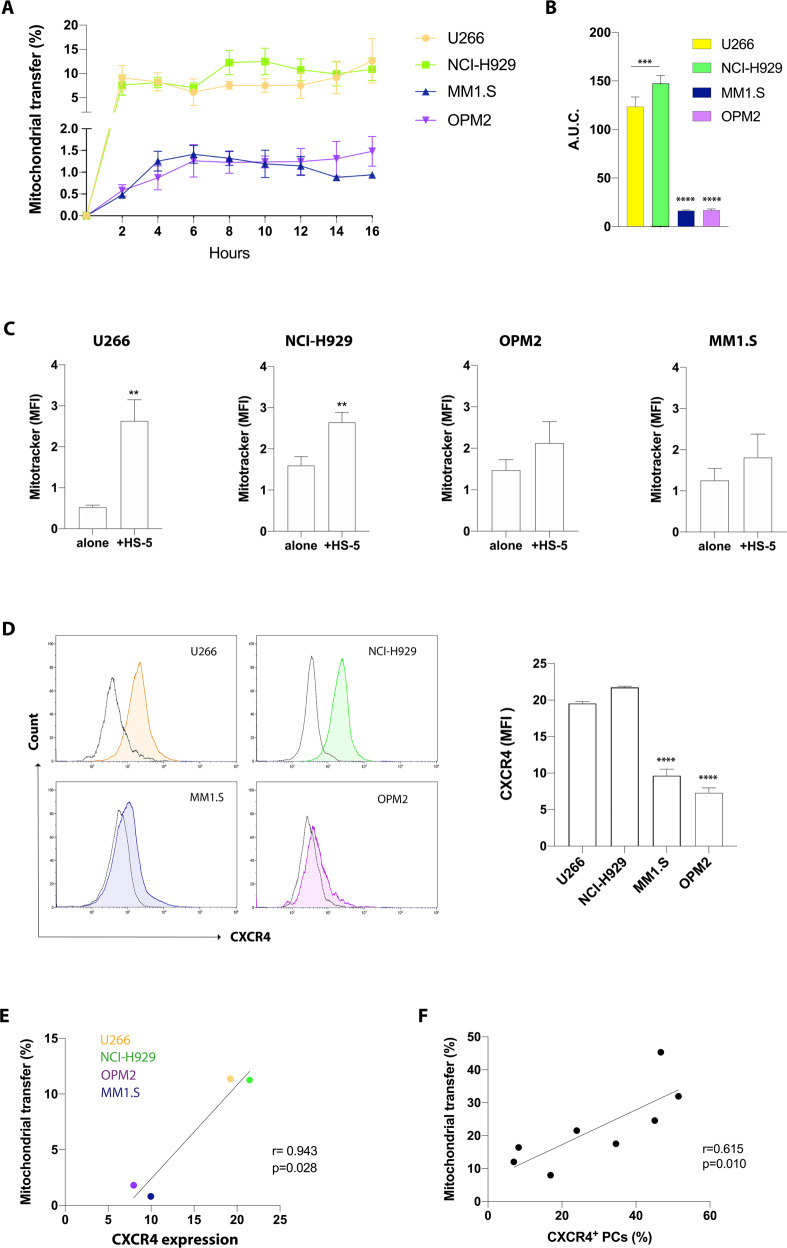
CXCR4 expression on PCs correlates with the percentage of transferred mitochondria. **A** Calcein AM labeled HMCLs were cultured alone or with MitoTracker red stained HS-5. Mitochondrial red fluorescence internalization was detected in HMCLs in time-lapse by using operetta. **B** Quantification of the area under the curve (A.U.C.) ± SD. **** vs U266 and NCI-H929 cell lines. **C** Flow cytometry analysis of mitochondrial red fluorescence internalized by HMCLs (CD45+ gated) cocultured with MitoTracker red labeled HS-5 cells for 24 h. **D** Flow cytometry analysis for CXCR4 expression of HMCLs. Representative histograms with the corresponding isotype control (dotted line) are shown. ****vs U266 and NCI-H929 cell lines. CXCR4 expression levels were correlated with the percentage of transferred mitochondria in HMCLs (**E**) and primary CD138+ cells (**F**). The data are presented as means ± SD of three independent experiments (**p < 0.01; ***p < 0.001; ****p < 0.0001).

CXCL12 and its receptor CXCR4 represent a critical communication axis between MSCs and tumor PCs to promote the expansion and colonization of myeloma PCs in the BM [31]. Therefore, we hypothesized that CXCR4 could be involved in mitochondrial transfer from MSCs to MM cells. We first labeled HMCLs with Calcein AM and then were cocultured with HS-5 cells stained with MitoTracker and we evaluated mitochondrial transfer in time-lapse by using operetta. Interestingly, differences in mitochondrial fluorescence internalization were detected in HMCLs, although a time-dependent increase was observed in all tested cell lines (Fig. 4A). Among the HMCLs, the percentage of mitochondria uptake was higher in U266 and NCI-H929 cells as demonstrated by an increase of the percentage of mitochondrial transfer respectively of about 9.5 ± 2.5 and 8.0 ± 2.07% in the first two hours from starting co-culture (*p* < 0.05). Mitochondria uptake was comparable between the two cell lines also after 16 h (12.1 ± 4.5% in U266 and 11.0 ± 2.33% in NCI-H929; *p* < 0.05 and *p* < 0.01 respectively). As mentioned above, an increase of mitochondria transfer was also observed in MM1.S and OMP2 cells, but with much lower magnitude (about 10 folds less) as compared to U266 and NCI-H929 cell lines. Particularly, MM1.S showed increased mitochondrial red fluorescence after 4 h from starting co-culture (1.3 ± 0.22%; *p* < 0.05) and the percentage of mitochondrial transfer decreased after 16 h (0.9 ± 0.05%; *p* < 0.01 versus t0). Consistently, OPM2 cells also showed low percentages of mitochondria uptake with a value of mitochondrial red fluorescence of about 1.5 ± 0.3% after 16 h from starting co-culture (*p* < 0.01 versus t0). The results were also expressed as total area under the curve (A.U.C.), and we found a significant lower value of the total A.U.C. in MM1.S and OPM2 than U266 and NCI-H929 cells (*p* < 0.0001; Fig. 4B). We also cocultured HMCLs with MitoTracker stained HS-5 to measure transferred mitochondria by flow cytometry. In accordance to the above data, we found that only U266 and NCI-H929 cells significantly increased MitoTracker-MFI values after 24 h coculture (from 0.53 ± 0.03 to 2.63 ± 0.42 and from 1.60 ± 0.21 to 2.64 ± 0.23, respectively; *p* < 0.01; Fig. 4C). No significant differences were observed in OMP2 and MM1.S.

We then analyzed CXCR4 surface expression in HMCLs by flow cytometry and observed higher CXCR4 levels in U266 and NCI-H929 (MFI: 19.6 ± 0.39 and 21.8 ± 0.26, respectively) as compared to MM1.S and OPM2 cell lines (MFI: 9.7 ± 1.52 and 7.3 ± 1.14; *p* < 0.0001; Fig. 4D). To investigate whether the degree of surface expression of CXCR4 influences mitochondrial transfer, we next correlated mitochondria uptake in HMCLs to their CXCR4 expression levels. Our data showed a strong correlation (*r* = 0.943; *p* = 0.028; Fig. 4E) which was confirmed also in primary myeloma cells (*n* = 8; *r* = 0.615; *p* = 0.01; Fig. 4F). This correlation between CXCR4 expression and the amount of transferred mitochondria was not dependent on effects of co-culture on CXCR4 expression in MM cells (Supplementary Fig. 6).

### CXCR4 inhibition reduces mitochondrial transfer from MSCs to myeloma PCs

**Fig. 5:**
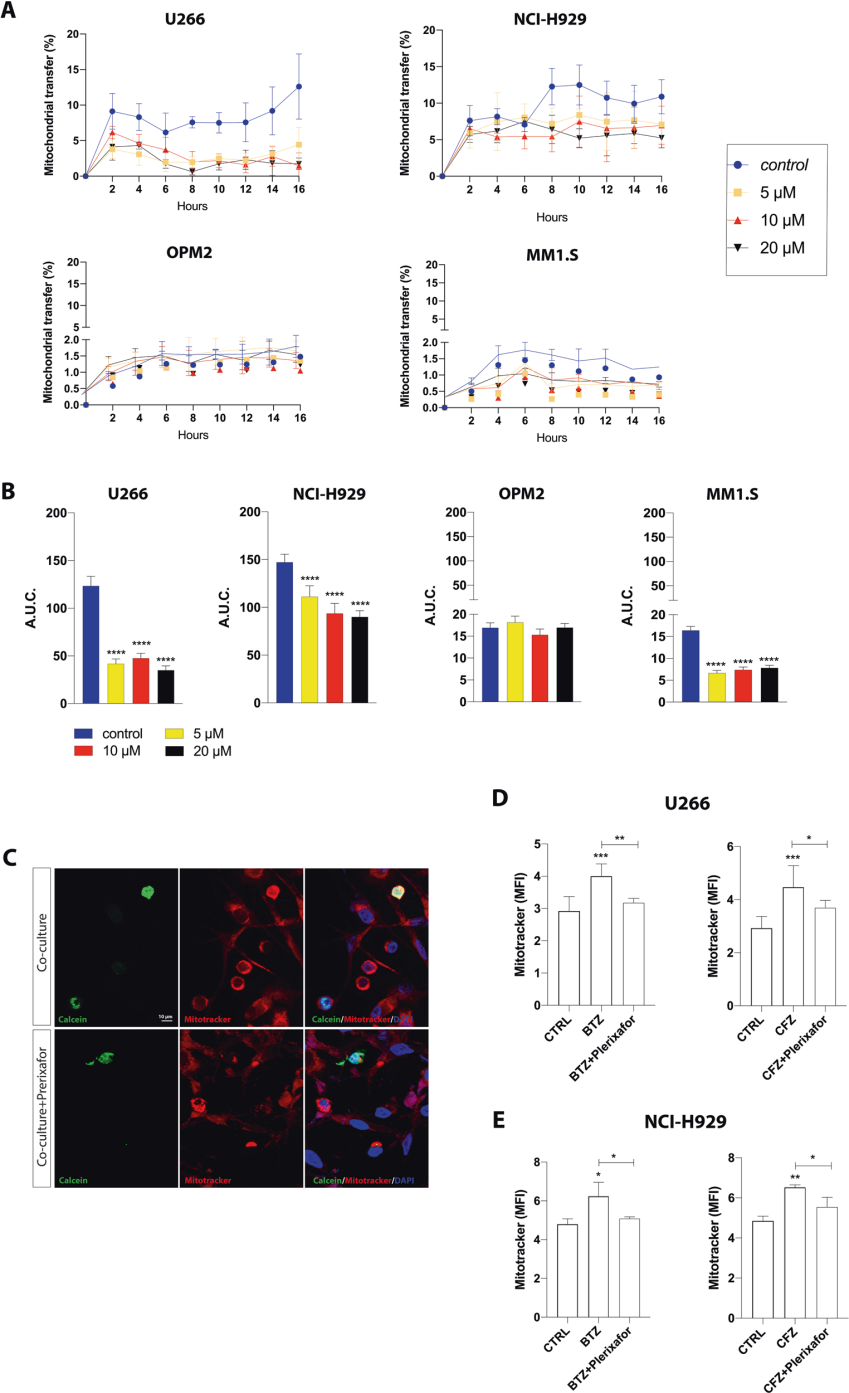
Inhibition of CXCR4 decreases mitochondrial transfer. **A** Plerixafor was added to the co-culture of Calcein AM stained HMCLs with MitoTracker red labeled HS-5 cells. Mitochondrial red fluorescence internalization in HMCLs was measured in time-lapse by operetta. **B** Quantification of the area under curve (A.U.C.) ± SD. **C** Representative confocal microscopy images show that 10 μM plerixafor decreased the amount of internalized MM-MSC cell-derived mitochondria (red) by Calcein AM-labeled CD138+ cells (green) after 24 h of co-culture. **D**, **E** After MitoTracker staining, HS5 cells were cultured with U266 or NCI-H929 in presence of 10 nM BTZ or 5 μM CFZ alone or in combination with 10 μM plerixafor. After 24 h mitochondrial transfer levels were assessed measuring MitoTracker red fluorescence by flow cytometry in MM cell lines. The data are presented as means ± SD of three independent experiments (*p < 0.05; **p < 0.01; ***p < 0.001; ****p < 0.0001).

To determine whether mitochondrial trafficking was mediated by CXCL12/CXCR4 axis, we cultured Calcein AM-stained HMCLs and MitoTracker-labeled HS-5 cells exposed to plerixafor, a CXCR4 receptor antagonist, at a final concentration of 5, 10, or 20 µM. The percentage of mitochondrial transfer was evaluated in time-lapse (Fig. 5A). 5 and 20 µM of plerixafor significantly reduced mitochondrial fluorescence intensity in U266 and HS-5 cells cocultures after 2 h, bringing the percentage of mitocondrial trasfer from 9.1 ± 2.5 to 3.8 ± 1.3% and 4.1 ± 1.9%, respectively (*p* < 0.0001 and *p* < 0.001). After 4 h of co-culture, 10 µM plerixafor was also able to significantly decrease mitochondrial transfer from 8.3 ± 1.9 to 4.6 ± 1.2% (*p* < 0.05). Lower levels of mitochondria uptake in presence of plerixafor was also observed in NCI-H929 cells after 8 h post plerixafor exposition (*p* < 0.05, *p* < 0.001, *p* < 0.01 at 5, 10 and 20 µM, respectively; Fig. 5A) and in MM1.S cells after 4 h (*p* < 0.0001 for all tested doses; Fig. 5A). Of note, mitochondrial transfer was not significantly affected by CXCR4 inhibitor in OPM2 cell line (Fig. 5A). Analysis of the overall A.U.C. confirmed the ineffectiveness of Plerixafor in inhibiting mitochondrial trafficking in OPM2 cells (Fig. 5B).

To confirm that inhibition of CXCR4 affects mitochondrial transfer, we also co-cultured U266-GFP cells with MitoTracker stained MM-MSCs to analyze mitochondria transfer by confocal-assisted microscopy. As shown in Fig. 5C, mitochondria uptake by myeloma PCs was decreased by plerixafor treatment.

Since it has been demonstrated that bortezomib (BTZ) exposure increases mitochondrial transfer from MSCs to myeloma PCs [12], we then repeated coculture experiments combining 10 µM plerixafor with the proteasome inhibitor (PI) BTZ or carfilzomib (CFZ). As expected, both 10 nM BTZ and 5 µM CFZ increased mitochondria uptake in MM cell lines (*p* < 0.001 for both BTZ or CFZ treated U266 versus control U266 *p* < 0.05 and *p* < 0.01 for BTZ or CFZ treated NCI-H929 versus control NCI-H929, respectively; Fig. 5D, E). The combination of plerixafor with BTZ or CFZ significantly decreased PI-induced mitochondrial transfer in U266 cell line (*p* < 0.01 and *p* < 0.05 for U266 treated with BTZ or CFZ combined with plerixafor versus U266 treated with BTZ or CFZ alone, respectively; Fig. 5D). These observations were confirmed in NCI-H929 cells, finding that plerixafor abolished mitochondrial transfer in co-treatment with either BTZ or CFZ (*p* < 0.05 for NCI-H929 treated with BTZ or CFZ combined with plerixafor versus NCI-H929 treated with BTZ or CFZ alone; Fig. 5E).

### Intracellular CXCR4 expression is higher in CD138+ cells from MM patients who failed to respond to bortezomib

**Fig. 6:**
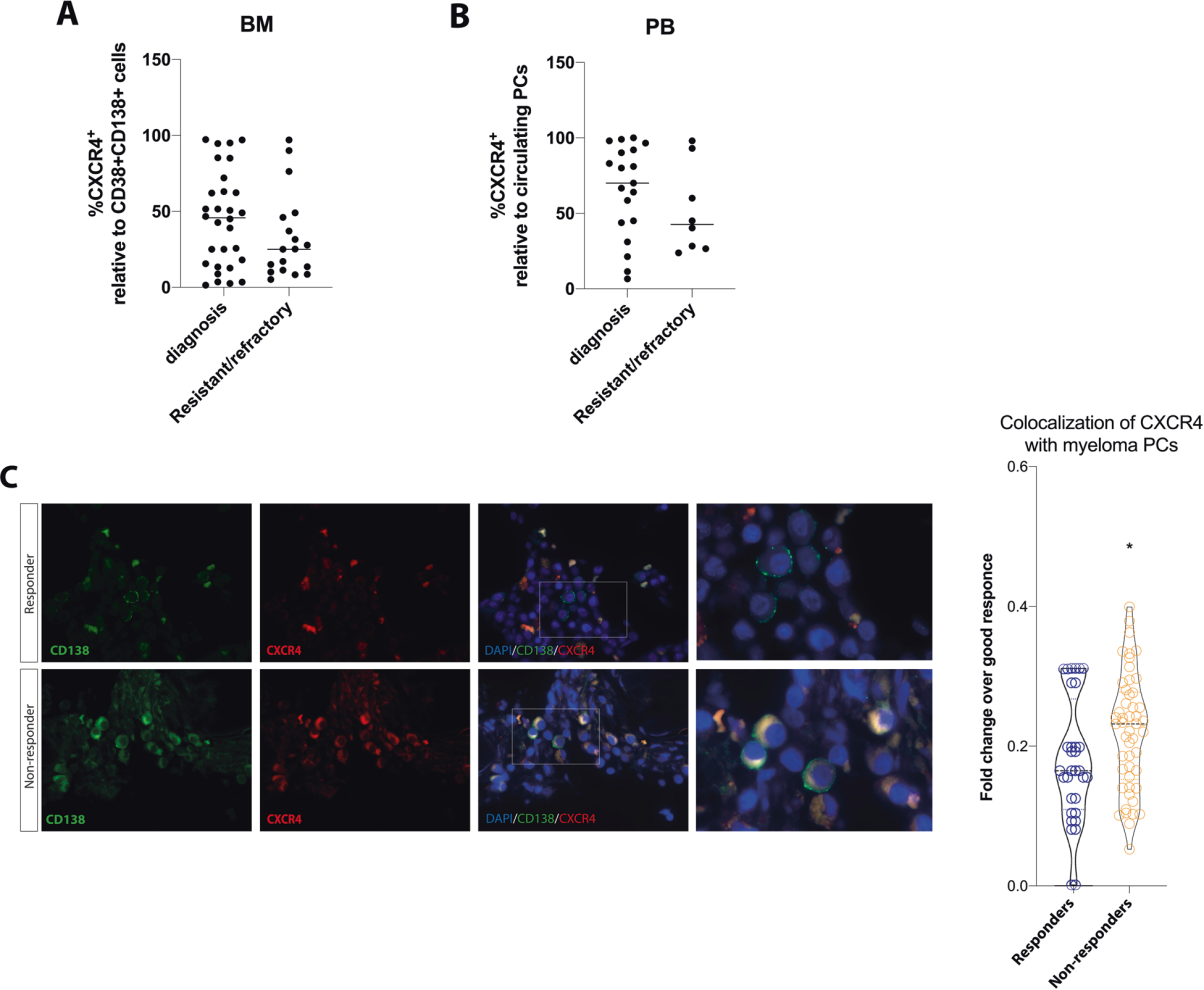
CXCR4 colocalizazion with CD138+ cells is higher in MM patients who failed to respond to BTZ. The amount of the percentage of CD138+CD38+CXCR4+ cells was assessed in BM (**A**) and PB (**B**) samples from MM at diagnosis (n = 30/19) and resistant/refractory patients (*n* = 18/8). **C** Analysis of CXCR4 colocalization with CD138+ cells in BM biopsy specimens from patients who responded to BTZ (*n* = 3) and patients who failed to respond (*n* = 3). The left panel shows representative pictures of a responder vs a non-responder patient. Scale bar: 10 μm. Bars indicate the standard error means (*p < 0.05).

CXCR4 inhibition enhances myeloma PCs sensitivity to therapeutic agents, including BTZ, through the disruption of their interaction with BM microenvironment [32]. Therefore, we assessed whether CXCR4 is differentially expressed on CD138^+^ PCs from MM patients at diagnosis and resistant/refractory patients by using flow cytometry. Our data revealed a wide range of surface CXCR4 expression on CD138^+^CD38^+^ cells, from low to high levels of expression in both analyzed subsets of MM patients. No significant difference in CXCR4 surface expression between PCs from BM of newly diagnosed (*n* = 30) and resistant/refractory (*n* = 18) patients was observed (Fig. 6A). Similar data were obtained analyzing CXCR4 expression on circulating PCs (19 patients at diagnosis and 8 resistant/refractory; Fig. 6B).

Interestingly, CXCR4 expression on PCs did not change between newly diagnosed and resistant/refractory patients, but a positive correlation was founded between response failure to PI therapy and increased colocalization of CXCR4 within CD138^+^ PCs in BM biopsy specimens. Indeed, MM patients who failed to respond to BTZ (*n* = 3) showed higher colocalization of CXCR4 with myeloma PCs compared with patients who responded (*n* = 3; *p* < 0.05 versus responders; Fig. 6C).

## Discussion

Mitochondrial transfer from non-malignant to malignant cells is recognized as a critical hallmark of tumor biology. In MM microenvironment mitochondria trafficking supports the oxidative metabolism of tumor PCs [12], which leads to increased production of ATP to support cancer progression and drug resistance [33–36]. Here, we demonstrated that MSCs within the protective microenvironment of MM transfer mitochondria to cancer cells, thus indicating that it represents a hallmark of MM-MSCs pro-tumorigenic phenotype. A growing body of evidence supports that the evolution of phenotype and function of MSCs from “naive” to tumor-derived MSCs is a complex process of tumor-mediated reprogramming necessary to satisfy the energy demand and tumor behavior of cancer cells in a hypoxic and nutrient-deficient microenvironment [37]. Reprogramming of MSCs by myeloma PCs induces pro-tumorigenic effects thus supporting cancer niche formation, malignant transformation, and tumor growth [3, 38], promotes resistance to anticancer drugs [39, 40] and maintains an immunosuppressive milieu [6, 41]. The alteration of the immunomodulatory properties of MM-MSCs could be dependent on their metabolic rewiring. Indeed, Liu and colleagues reported that MSC metabolic pathways remodeling towards glycolysis is required to sustain their immunosuppressive activities [10]. Compared to healthy MSCs, MM-MSCs had a lower NAD^+^/NADH ratio associated to higher intracellular lactate concentration indicating a more glycolytic metabolism. Our data are consistent with the strong evidence that MM-MSCs actively contribute to immune escape mechanisms that take place in MM microenvironment. The consequent reduced reliance on oxidative metabolism of MM-MSCs may also contribute to their increased ability to transfer mitochondria.

CXCL12/CXCR4 axis plays a key role in the homing of normal and MM cells at BM level [40]. CXCL12 is a pivotal regulator of the tumor microenvironment regulating multiple oncogenic processes such as angiogenesis, osteoclastogenesis, or tumor cell migration and adhesion to stromal cells [42]. Moreover, CXCL12 supports MM growth through the induction of interleukin-6 (IL-6) and vascular endothelial growth factor (VEGF) secretion and increases invasion and matrix metalloproteinase (MMP) secretion in myeloma PCs [32, 43]. CXCL12 expression is higher in myeloma BM compared with healthy BM [44] and cancer-associated fibroblasts (CAFs) from patients with active MM produce higher levels of CXCL12 compared from non-active MM, MGUS, and healthy subjects [45]. Consistently, our data showed increased expression of CXCL12 in MM-MSCs compared with HC-MSCs. We also demonstrated that increased PC-mediated CXCL12 expression is regulated by CX43 channels throughout cell-to-cell communication [46]. Indeed, increased CXCL12 expression in HS-5 cells after coculture with PCs was significantly decreased by selective inhibition of CX43-based gap junctions. This is consistent with previous findings demonstrating that the secretion of CXCL12 by MSCs is dependent on CX43 [29] and CX43 expression in MM-MSCs favors the interactions with PCs regulating their adhesion and migration [19]. Since there is evidence that mitochondrial transfer also occurs via heterocellular interactions [47] and can be mediated by gap junctional channels formed by CX43 [48, 49], we further investigated its involvement in mitochondria trafficking in myeloma microenvironment. We found that inhibition of CX43-based gap junctions almost completely inhibited mitochondrial transfer from HS-5 to MM cells suggesting that not only MM-MSCs transfer more mitochondria, but also PCs promote this trafficking inducing upregulation of CX43 expression. Given that MM PCs induced increased CX43 and CXCL12 colocalization in HS-5 cells, we supposed that CXCL12/CXCR4 signaling could regulate mitochondria trafficking throughout this axis. For this reason, we analyzed the kinetic of mitochondria uptake of several HMCLs and related their CXCR4 expression with the percentage of transferred mitochondria. Our data demonstrated that HMCLs with higher expression of CXCR4 had also higher percentage of transferred mitochondria both in time lapse and flow cytometry. The correlation between CXCR4 expression and the percentage of mitochondria uptake in HMCLs was also confirmed in primary myeloma PCs. Furthermore, plerixafor, a selective inhibitor of CXCR4, significantly reduced mitochondrial transfer from MSCs to myeloma PCs further establishing mechanistically that CXCR4/CXCL12 is directly involved in mitochondrial trafficking. Our data further pointed out that CXCR4/CXCL12 pathway may serve as a new strategy to target myeloma niche. Our in vitro results are further corroborated in clinical trials involving MM patients [50]. To this regard, a phase I/II trial reports that BTZ/plerixafor combination achieves a strong response rate even in relapsed/refractory patients previously treated with BTZ [51]. In particular, the inhibition of CXCR4 disrupts MM cell adhesion to BM MSC mobilizing myeloma PCs into circulation and increasing BTZ-induced apoptosis in vivo [32]. Therefore, we investigated whether the combination of plerixafor with BTZ or CFZ interferes with mitochondrial transfer from MSCs to PCs. Interestingly, we found that the proteasome inhibitors promoted mitochondrial transfer while their combination with plerixafor inhibited mitochondria trafficking. It is known that proteasomal inhibition compromises mitochondrial respiration in MM cells which need higher amount of ATP for chaperone-driven protein folding and to protect themselves against PI-induced ER-stress [52].

According to the latter evidence, we found an increased amount of transferred mitochondria in cocultures after exposure to PIs and hypothesize that plerixafor-induced sensitization to therapeutic agents including BTZ [32] could be in part dependent by blocking of metabolic coupling of MSCs to tumor PCs mediated by mitochondria trafficking.

Finally, we evaluated whether CXCR4 on myeloma PCs was differently expressed in resistant/refractory MM patients compared to MM ones at diagnosis. No significant differences were observed neither on MM cells from BM nor on circulating PCs with a wide range of surface CXCR4 expression in both groups of patients. However, the amount of CXCR4 on MM cells strives for a decrease in resistant/refractory patients both in BM and PB. In agreement, higher CXCL12 levels in BM have been associated to lower CXCR4 expression on the surface of MM cells and are related with early poor survival outcomes for patients [53]. Interestingly, evaluating intracellular expression of CXCR4 in myeloma PCs from BM biopsy specimens, we found higher intracellular CXCR4 levels in CD138+ cells of patients who failed to respond to BTZ compared with patients who responded, indicating that CXCR4 may influence response to chemotherapy in MM patients.

**Fig. 7:**
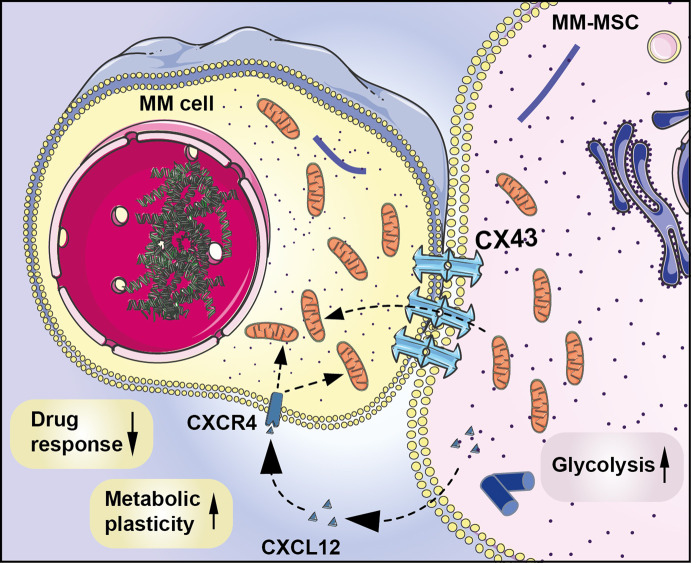
MSC plasticity in MM microenvironment. Pro-tumor phenotype of MM-MSCs is associated to a metabolic rewiring and an increased ability to donate mitochondria. Furthermore, myeloma PCs increase the expression of CX43 and CXCL12 proteins in stromal cells with consequent activation of the corresponding receptor CXCR4, which favors mitochondrial uptake from MSCs. The high mitochondrial trafficking in MM microenvironment supports tumor to escape PI-induced intracellular oxidative stress and mitochondrial damage.

In conclusion, we have shown that MM-MSCs have increased ability to deliver mitochondria, probably on the basis of their metabolic rewiring. Furthermore, tumor PCs increase the expression of CX43 in MSCs leading to an increased levels of CXCL12 and stimulation of its corresponding receptor expressed on MM cells (Fig. 7). The resulting CX43/CXCL12/CXCR4 interplay enhances mitochondrial trafficking from MSCs to myeloma PCs and can protect cancer cells against anti-myeloma agents. Understanding pro-tumorigenic phenotype of MSCs and mechanisms of adhesion and heterocellular communication favoring their interaction with cancer PCs, will allow to manipulate critical pathways, including CXCL12/CXCR4 axis, thus improving disease outcome.

## Supplementary information

Supplementary figures

## References

[1] Gulla A, Anderson KC (2020). Multiple myeloma: The (r)evolution of current therapy and a glance into future. Haematologica.

[2] Camiolo G, Barbato A, Giallongo C, Vicario N, Romano A, Parrinello NL (2020). Iron regulates myeloma cell/macrophage interaction and drives resistance to bortezomib. Redox Biol.

[3] Giannakoulas N, Ntanasis-Stathopoulos I, Terpos E (2021). The role of marrow microenvironment in the growth and development of malignant plasma cells in multiple myeloma. Int J Mol Sci.

[4] Schinke C, Qu P, Mehdi SJ, Hoering A, Epstein J, Johnson SK (2018). The pattern of mesenchymal stem cell expression is an independent marker of outcome in multiple myeloma. Clin Cancer Res.

[5] De Veirman K, Wang J, Xu S, Leleu X, Himpe E, Maes K (2016). Induction of miR-146a by multiple myeloma cells in mesenchymal stromal cells stimulates their pro-tumoral activity. Cancer Lett.

[6] Giallongo C, Tibullo D, Parrinello NL, La Cava P, Di Rosa M, Bramanti V (2016). Granulocyte-like myeloid derived suppressor cells (G-MDSC) are increased in multiple myeloma and are driven by dysfunctional mesenchymal stem cells (MSC). Oncotarget.

[7] Giallongo C, Tibullo D, Camiolo G, Parrinello NL, Romano A, Puglisi F, et al. TLR4 signaling drives mesenchymal stromal cells commitment to promote tumor microenvironment transformation in multiple myeloma. Cell Death Dis. 2019;10:704. 10.1038/s41419-019-1959-5 PMC6754430 31541083

[8] Lemaitre L, DoSouto Ferreira L, Joubert MV, Avet-Loiseau H, Martinet L, Corre J, et al. Imprinting of mesenchymal stromal cell transcriptome persists even after treatment in patients with multiple myeloma. Int J Mol Sci. 2020;21:3854. 10.3390/ijms21113854 PMC7312921 32481768

[9] de Jong MME, Kellermayer Z, Papazian N, Tahri S, Hofste Op Bruinink D, Hoogenboezem R (2021). The multiple myeloma microenvironment is defined by an inflammatory stromal cell landscape. Nat Immunol.

[10] Liu Y, Yuan X, Munoz N, Logan TM, Ma T (2019). Commitment to aerobic glycolysis sustains immunosuppression of human mesenchymal stem cells. Stem Cells Transl Med.

[11] Marlein CR, Zaitseva L, Piddock RE, Robinson SD, Edwards DR, Shafat MS (2017). NADPH oxidase-2 derived superoxide drives mitochondrial transfer from bone marrow stromal cells to leukemic blasts. Blood.

[12] Marlein CR, Piddock RE, Mistry JJ, Zaitseva L, Hellmich C, Horton RH (2019). CD38-driven mitochondrial trafficking promotes bioenergetic plasticity in multiple myeloma. Cancer Res.

[13] Moschoi R, Imbert V, Nebout M, Chiche J, Mary D, Prebet T (2016). Protective mitochondrial transfer from bone marrow stromal cells to acute myeloid leukemic cells during chemotherapy. Blood.

[14] Wang J, Liu X, Qiu Y, Shi Y, Cai J, Wang B (2018). Cell adhesion-mediated mitochondria transfer contributes to mesenchymal stem cell-induced chemoresistance on T cell acute lymphoblastic leukemia cells. J Hematol Oncol.

[15] Pinto G, Saenz-de-Santa-Maria I, Chastagner P, Perthame E, Delmas C, Toulas C (2021). Patient-derived glioblastoma stem cells transfer mitochondria through tunneling nanotubes in tumor organoids. Biochem J.

[16] Mahrouf-Yorgov M, Augeul L, Da Silva CC, Jourdan M, Rigolet M, Manin S (2017). Mesenchymal stem cells sense mitochondria released from damaged cells as danger signals to activate their rescue properties. Cell Death Differ.

[17] Ahmad T, Mukherjee S, Pattnaik B, Kumar M, Singh S, Kumar M (2014). Miro1 regulates intercellular mitochondrial transport & enhances mesenchymal stem cell rescue efficacy. EMBO J.

[18] Islam MN, Das SR, Emin MT, Wei M, Sun L, Westphalen K (2012). Mitochondrial transfer from bone-marrow-derived stromal cells to pulmonary alveoli protects against acute lung injury. Nat Med.

[19] Zhang X, Sun Y, Wang Z, Huang Z, Li B, Fu J (2015). Up-regulation of connexin-43 expression in bone marrow mesenchymal stem cells plays a crucial role in adhesion and migration of multiple myeloma cells. Leuk Lymphoma.

[20] Lazzarino G, Amorini AM, Fazzina G, Vagnozzi R, Signoretti S, Donzelli S (2003). Single-sample preparation for simultaneous cellular redox and energy state determination. Anal Biochem.

[21] Amorini AM, Nociti V, Petzold A, Gasperini C, Quartuccio E, Lazzarino G (2014). Serum lactate as a novel potential biomarker in multiple sclerosis. Biochim Biophys Acta.

[22] Xu Y, Lu S (2015). Transforming growth factor-beta1-induced epithelial to mesenchymal transition increases mitochondrial content in the A549 non-small cell lung cancer cell line. Mol Med Rep.

[23] Quiros PM, Goyal A, Jha P, Auwerx J (2017). Analysis of mtDNA/nDNA ratio in mice. Curr Protoc Mouse Biol.

[24] Vicario N, Bernstock JD, Spitale FM, Giallongo C, Giunta MAS, Li Volti G, et al. Clobetasol modulates adult neural stem cell growth via canonical hedgehog pathway activation. Int J Mol Sci. 2019;20:1991. 10.3390/ijms20081991 PMC6514872 31018557

[25] Camiolo G, Tibullo D, Giallongo C, Romano A, Parrinello NL, Musumeci G, et al. alpha-Lipoic acid reduces iron-induced toxicity and oxidative stress in a model of iron overload. Int J Mol Sci. 2019;20:609. 10.3390/ijms20030609 PMC6387298 30708965

[26] de Almeida MJ, Luchsinger LL, Corrigan DJ, Williams LJ, Snoeck HW (2017). Dye-independent methods reveal elevated mitochondrial mass in hematopoietic stem cells. Cell Stem Cell.

[27] Norris RP (2021). Transfer of mitochondria and endosomes between cells by gap junction internalization. Traffic.

[28] Ren D, Zheng P, Zou S, Gong Y, Wang Y, Duan J, et al. GJA1-20K Enhances mitochondria transfer from astrocytes to neurons via Cx43-TnTs after traumatic brain injury. Cell Mol Neurobiol. 2021. 10.1007/s10571-021-01070-x. 10.1007/s10571-021-01070-x PMC11421752 33728536

[29] Schajnovitz A, Itkin T, D’Uva G, Kalinkovich A, Golan K, Ludin A (2011). CXCL12 secretion by bone marrow stromal cells is dependent on cell contact and mediated by connexin-43 and connexin-45 gap junctions. Nat Immunol.

[30] Vicario N, Calabrese G, Zappala A, Parenti C, Forte S, Graziano ACE (2017). Inhibition of Cx43 mediates protective effects on hypoxic/reoxygenated human neuroblastoma cells. J Cell Mol Med.

[31] Guo F, Wang Y, Liu J, Mok SC, Xue F, Zhang W (2016). CXCL12/CXCR4: A symbiotic bridge linking cancer cells and their stromal neighbors in oncogenic communication networks. Oncogene.

[32] Azab AK, Runnels JM, Pitsillides C, Moreau AS, Azab F, Leleu X (2009). CXCR4 inhibitor AMD3100 disrupts the interaction of multiple myeloma cells with the bone marrow microenvironment and enhances their sensitivity to therapy. Blood.

[33] Barbato A, Scandura G, Puglisi F, Cambria D, La Spina E, Palumbo GA (2020). Mitochondrial bioenergetics at the onset of drug resistance in hematological malignancies: An overview. Front Oncol.

[34] Cohen YC, Zada M, Wang SY, Bornstein C, David E, Moshe A (2021). Identification of resistance pathways and therapeutic targets in relapsed multiple myeloma patients through single-cell sequencing. Nat Med.

[35] Giallongo C, Tibullo D, Puglisi F, Barbato A, Vicario N, Cambria D, et al. Inhibition of TLR4 signaling affects mitochondrial fitness and overcomes bortezomib resistance in myeloma plasma cells. Cancers. 2020;12:1999. 10.3390/cancers12081999 PMC7463509 32707760

[36] Tibullo D, Giallongo C, Romano A, Vicario N, Barbato A, Puglisi F, et al. Mitochondrial functions, energy metabolism, and protein glycosylation are interconnected processes mediating resistance to bortezomib in multiple myeloma cells. Biomolecules. 2020;10:696. 10.3390/biom10050696 PMC7277183 32365811

[37] Nwabo Kamdje AH, Seke Etet PF, Simo Tagne R, Vecchio L, Lukong KE, Krampera M (2020). Tumor microenvironment uses a reversible reprogramming of mesenchymal stromal cells to mediate pro-tumorigenic effects. Front Cell Dev Biol.

[38] Tibullo D, Longo A, Vicario N, Romano A, Barbato A, Di Rosa M, et al. Ixazomib improves bone remodeling and counteracts sonic hedgehog signaling inhibition mediated by myeloma cells. Cancers*.* 2020;12:323. 10.3390/cancers12020323 PMC7073172 32019102

[39] Huang J, Huang LQ, He HS, Yan J, Huang C, Wang R (2020). Overexpression of heme oxygenase-1 in bone marrow stromal cells promotes multiple myeloma resistance through the JAK2/STAT3 pathway. Life Sci.

[40] Ren Z, Lantermans H, Kuil A, Kraan W, Arenzana-Seisdedos F, Kersten MJ (2021). The CXCL12gamma chemokine immobilized by heparan sulfate on stromal niche cells controls adhesion and mediates drug resistance in multiple myeloma. J Hematol Oncol.

[41] Liu Z, Mi F, Han M, Tian M, Deng L, Meng N (2021). Bone marrow-derived mesenchymal stem cells inhibit CD8(+) T cell immune responses via PD-1/PD-L1 pathway in multiple myeloma. Clin Exp Immunol.

[42] Bouyssou JM, Ghobrial IM, Roccaro AM (2016). Targeting SDF-1 in multiple myeloma tumor microenvironment. Cancer Lett.

[43] Parmo-Cabanas M, Molina-Ortiz I, Matias-Roman S, Garcia-Bernal D, Carvajal-Vergara X, Valle I (2006). Role of metalloproteinases MMP-9 and MT1-MMP in CXCL12-promoted myeloma cell invasion across basement membranes. J Pathol.

[44] Alsayed Y, Ngo H, Runnels J, Leleu X, Singha UK, Pitsillides CM (2007). Mechanisms of regulation of CXCR4/SDF-1 (CXCL12)-dependent migration and homing in multiple myeloma. Blood.

[45] Frassanito MA, Rao L, Moschetta M, Ria R, Di Marzo L, De, Luisi A (2014). Bone marrow fibroblasts parallel multiple myeloma progression in patients and mice: in vitro and in vivo studies. Leukemia.

[46] Vicario N, Zappala A, Calabrese G, Gulino R, Parenti C, Gulisano M (2017). Connexins in the central nervous system: Physiological traits and neuroprotective targets. Front Physiol.

[47] Peruzzotti-Jametti L, Bernstock JD, Willis CM, Manferrari G, Rogall R, Fernandez-Vizarra E, et al. Neural stem cells traffic functional mitochondria via extracellular vesicles. PLoS Biol. 2021;19:e3001166. 10.1371/journal.pbio.3001166 PMC8055036 33826607

[48] Golan K, Singh AK, Kollet O, Bertagna M, Althoff MJ, Khatib-Massalha E (2020). Bone marrow regeneration requires mitochondrial transfer from donor Cx43-expressing hematopoietic progenitors to stroma. Blood.

[49] Yao Y, Fan XL, Jiang D, Zhang Y, Li X, Xu ZB (2018). Connexin 43-mediated mitochondrial transfer of iPSC-MSCs alleviates asthma inflammation. Stem Cell Rep.

[50] Ghobrial IM, Liu CJ, Redd RA, Perez RP, Baz R, Zavidij O (2020). A phase Ib/II trial of the first-in-class anti-CXCR4 antibody Ulocuplumab in combination with lenalidomide or bortezomib plus dexamethasone in relapsed multiple myeloma. Clin Cancer Res.

[51] Ghobrial IM, Liu CJ, Zavidij O, Azab AK, Baz R, Laubach JP (2019). Phase I/II trial of the CXCR4 inhibitor plerixafor in combination with bortezomib as a chemosensitization strategy in relapsed/refractory multiple myeloma. Am J Hematol.

[52] Besse L, Besse A, Mendez-Lopez M, Vasickova K, Sedlackova M, Vanhara P (2019). A metabolic switch in proteasome inhibitor-resistant multiple myeloma ensures higher mitochondrial metabolism, protein folding, and sphingomyelin synthesis. Haematologica.

[53] Vandyke K, Zeissig MN, Hewett DR, Martin SK, Mrozik KM, Cheong CM (2017). HIF-2alpha promotes dissemination of plasma cells in multiple myeloma by regulating CXCL12/CXCR4 and CCR1. Cancer Res.

