# Supplementary material for: CXCL12/CXCR4 axis supports mitochondrial trafficking in tumor myeloma microenvironment
Source: Oncogenesis. 2022 Jan 21;11(1):6. doi: 10.1038/s41389-022-00380-z (PMC8782911; doi:10.1038/s41389-022-00380-z)
Supplement: Supplementary file 1 — Supplementary figures [file 41389_2022_380_MOESM1_ESM.docx]

**SUPPLEMENTARY FIGURES**

**
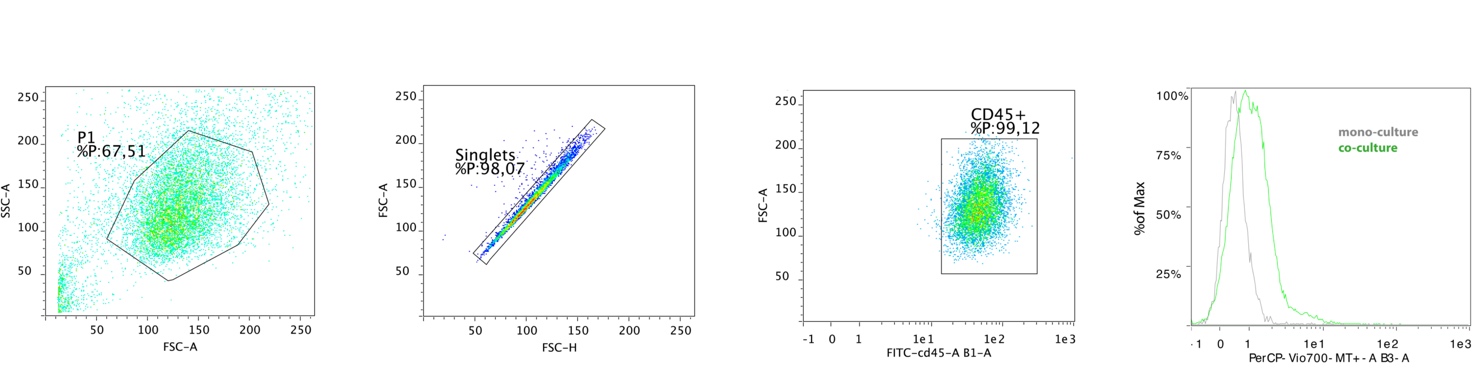
**

**Supplementary Figure 1**

**Gating strategy for flow cytometry based mitochondrial transfer experiments.** After 24h co-culture with MitoTracker stained HS-5, MM cells were collected and labelled using anti-human CD45-FITC antibody (clone J33, BECKMAN COULTER). Exclusion of doublets was confirmed using the FSH-A and FSH-H plot. The MitoTracker red mean fluorescence in B3 of the gated MM cells (CD45 positive cells) was used to asses mitochondrial transfer.


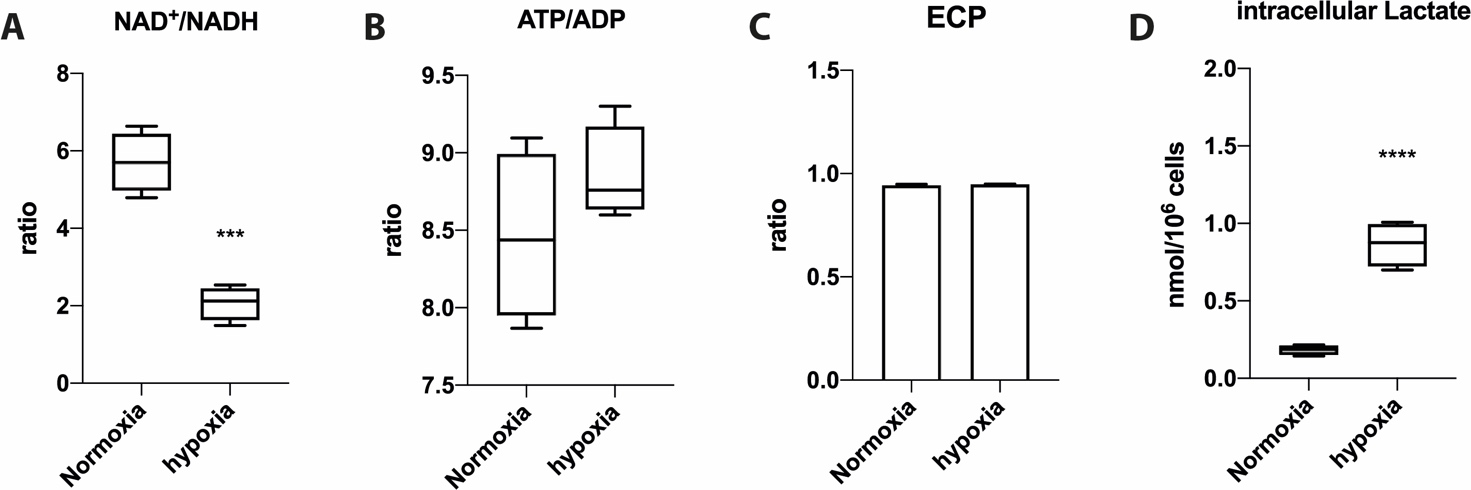


**Supplementary Figure 2. Metabolic changes induced by hypoxia in HS-5 cells resemble the metabolic rewiring of MM-MSCs. A-C.** NAD^+^, NADH, ATP and ADP concentrations were calculated by HPLC analysis in deproteinized HS-5 cells cultured for 24h in normoxic or hypoxic conditions (1.0% O_2_). ECP was calculated as follows: ATP+1/2ADP/ATP+ADP+AMP. **D.** Intracellular lactate concentration was measured by enzymatic assay. The data are presented as means ± SD of three independent experiments (***p<0.001; ****p<0.0001).


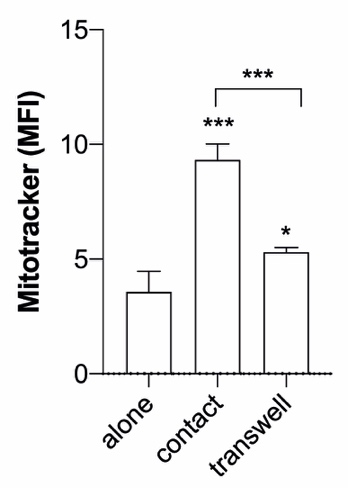


**Supplementary Figure 3. Blocking cell-to-cell contact decreases mitochondrial transfer from MSCs to myeloma PCs.** U266 cells were co-cultured with MitoTracker labelled HS-5 cell line in regular wells or in a transwell assay (inserts with 0.4-μm pores) to prevent direct cell-cell contact. After 24h, MitoTracker red fluorescence was measured in U266 cells (CD45+ gated) by flow cytometry. U266 cells cultured alone were used as control. The data are presented as means ± SD of three independent experiments (*p<0.05; ***p<0.001).


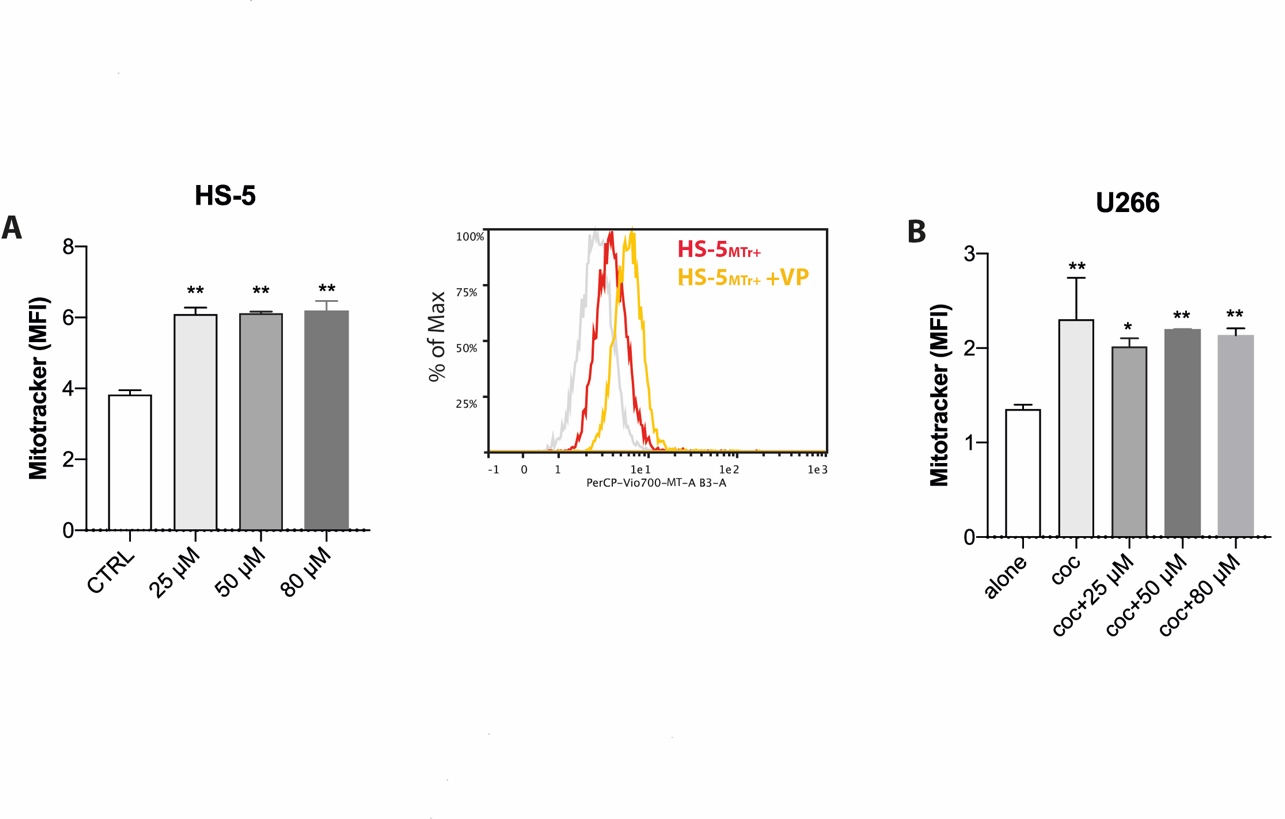


**Supplementary Figure 4. Efflux pumps of MSCs does not influence mitochondrial trafficking between stromal and MM cells. A.** MitoTracker labelled HS-5 cells were exposed to 25, 50 and 80μM of VP. After 24h, MitoTracker red fluorescence was measured by flow cytometry. A representative flow cytometry histogram shows the comparison of MitoTracker red fluorescence of labelled HS-5 cells in absence or presence of VP (50μM) for 24h. Gray histogram shows unstained cells. **B.** U266 cells were cocultured with MitoTracker labelled HS-5 cells in presence or not of VP for 24h. MitoTracker red fluorescence was measured in U266 cells (CD45+ gated) by flow cytometry and MM cells cultured alone were used as control. The data are presented as means ± SD of three independent experiments (*p<0.05; **p<0.01).


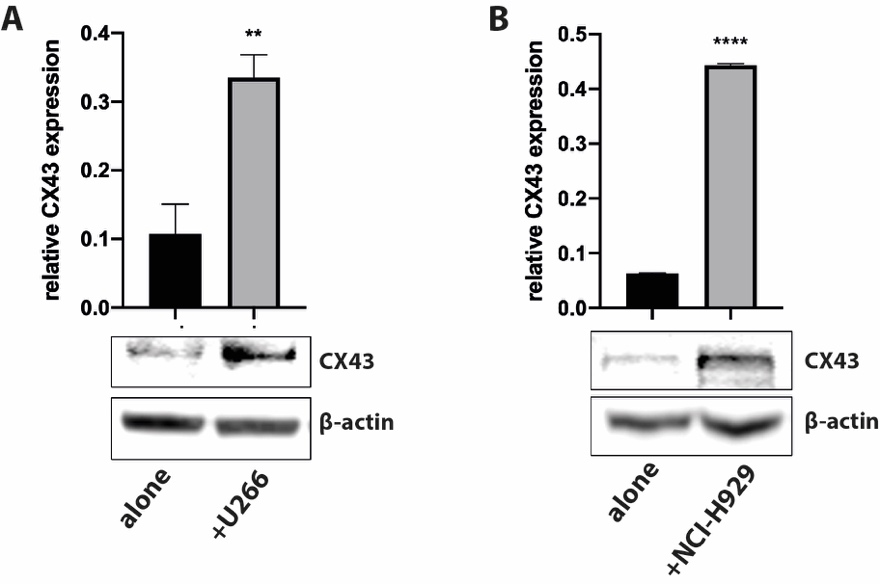


**Supplementary Figure 5. MM cells increases CX43 expression in HS-5 cell line.** After 24h co-culture with U266 (A) or NCI-H929 (B), HS-5 cells were analyzed for expression of CX43 by using western blot. β-actin protein was used as total protein loading reference. For analysis, the optical density of the bands was measured using Scion Image software. The data are presented as means ± SD of three independent experiments (**p<0.01; ****p<0.0001).


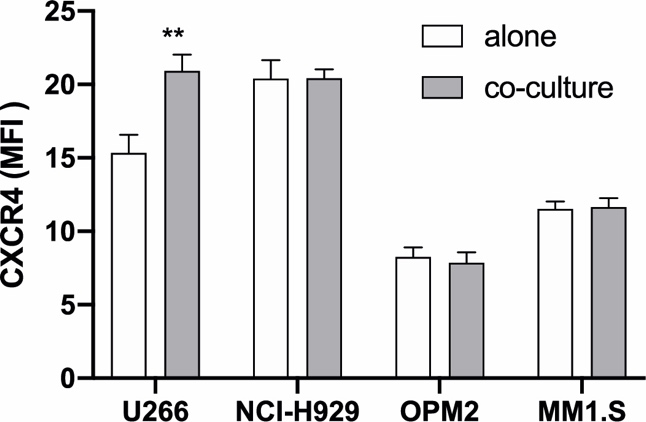


**Supplementary Figure 6. Effect of co-culture on CXCR4 expression in HMCLs.** Expression of CXCR4 was evaluated in MM cell lines after 24h co-culture with HS-5 cells by flow cytometry. The data are presented as means ± SD of three independent experiments (**p<0.01).
